# Pharmacokinetics and thermal anti-nociceptive effects of oral morphine in horses

**DOI:** 10.3389/fvets.2024.1461648

**Published:** 2024-09-17

**Authors:** Heather K. Knych, Stacy J. Steinmetz, Megan L. Traynham, Daniel S. McKemie, Philip H. Kass

**Affiliations:** ^1^K.L. Maddy Equine Analytical Chemistry Laboratory (Pharmacology Section), School of Veterinary Medicine, University of California, Davis, Davis, CA, United States; ^2^Department of Molecular Biosciences, School of Veterinary Medicine, University of California, Davis, Davis, CA, United States; ^3^Department of Population Health and Reproduction, School of Veterinary Medicine, University of California, Davis, Davis, CA, United States

**Keywords:** horse, morphine, oral, multiple doses, thermal nociception, pharmacokinetics, pharmacodynamics

## Abstract

**Introduction:**

Morphine is an effective analgesic in horses, however, IV administration at therapeutic doses has been shown to produce dose-dependent neuroexcitation and unwanted gastrointestinal effects. The analgesic effects of morphine have, at least in part, been attributed to the morphine-6-glucuronide (M6G) metabolite. Oral administration to horses results in comparable M6G concentrations to that achieved following IV administration of a therapeutic dose without the adverse effects. The anti-nociceptive effects have not yet been reported. In the current study the thermal anti-nociceptive effects of single and multiple oral doses of morphine were assessed.

**Methods:**

Six horses received a single 0.2 mg/kg IV dose of morphine and multiple oral doses of 0.8 mg/kg morphine every 12 h for 4.5 days. Blood samples were collected throughout administration, morphine, and metabolite concentrations determined and pharmacokinetic analysis performed. Drug related behavior and physiologic responses were recorded. Response to noxious stimuli was evaluated by determining thermal threshold latency in response to the application of heat.

**Results:**

The maximum concentrations of M6G were higher following oral administration compared to IV and the combined morphine and M6G concentrations exceeded that of IV administration starting at 2 h. Oral administration of 0.8 mg/kg morphine provided and maintained comparable anti-nociception effects to IV morphine with less adverse effects, following single and multiple doses. Morphine was well tolerated following oral administration with less excitation and minimal effects on gastrointestinal borborygmi scores compared to IV administration.

**Discussion:**

Results of the current study warrant further investigation of the anti-nociceptive effects of oral morphine administration to horses.

## 1 Introduction

Morphine is a potent analgesic used commonly in humans and to a lesser extent in small animal medicine. Dose-dependent undesirable effects, including neuroexcitation and gastrointestinal effects (i.e., decreased motility) at therapeutic concentrations have limited its use in horses (1–5). In many species, including horses, morphine is metabolized to morphine-6-glucuronide (M6G) and morphine-3-glucuronide (M3G) (1, 2, 6). Morphine-3-glucuronide reportedly can lead to dose dependent behavioral excitation in some species (7–9), however, M3G administration to a small group of horses (*n* = 4) did not appear to elicit a neuroexcitatory effect, suggesting that perhaps morphine is responsible for this effect in horses (10). A portion of the analgesic effect of morphine in humans and laboratory animal species has been attributed to the, M6G metabolite (11–15). Interestingly, in lab animal species, M6G appears to be devoid of many of the adverse effects noted with morphine administration (13). In a study conducted previously it was demonstrated that M6G administration resulted in a thermal anti-nociceptive effect following intravenous (IV) administration to horses, without significant adverse effects (16). The number of animals studied (*n* = 7) was small, however, these results suggest that, similar to other species, M6G elicits a thermal anti-nociceptive affect in horses (16). Although M6G has been pursued as a therapeutic agent in human medicine, it is not currently commercially available and the cost to synthesize this compound for administration to horses is cost prohibitive. In a more recent study the pharmacokinetics and metabolism of morphine, including the concentration of the metabolite relative to the concentration of morphine (M6G or M3G: morphine), following oral administration to horses was described (6). The metabolite to morphine ratio is a good indicator of the relative amount of morphine that undergoes biotransformation to a particular metabolite. Higher concentrations of M6G were reached following oral administration of 0.8 mg/kg morphine compared to IV administration of 0.2 mg/kg. Higher concentrations of the metabolite are presumably due to the high susceptibility of morphine to first pass glucuronidation and increased conversion to the presumed active metabolite. Although we demonstrated that higher morphine and M6G concentrations could be achieved following oral administration to horses, and in humans the relative contribution of M6G to the overall analgesic effect of morphine following oral administration is reportedly 96.6% (17), the anti-nociceptive effects of morphine following administration by this route have yet to be described in horses.

In humans, oral morphine is more effective following multiple doses (18). Using pharmacokinetic and simulation modeling, one group of investigators determined that administration of morphine to humans at 12 h intervals for 5 days results in accumulation of the active metabolite, M6G at the effector site without evidence of accumulation in plasma (19). Furthermore, they concluded that accumulation of M6G at the effector site after chronic oral dosing of morphine, resulted in a 2-fold higher concentration of M6G at the site, leading to a 4-fold higher potency compared to a single oral dose (19). There are several studies describing the pharmacokinetics and pharmacodynamics of morphine in horses following a single dose (1, 2), as well as evidence of entry of M6G into the CNS following IV administration of the metabolite to horses (20). However, there are currently no published studies describing the pharmacokinetics or anti-nociceptive effects of oral morphine following single or multiple administrations to horses.

In the current study, we hypothesized that oral administration of 0.8 mg/kg morphine would result in higher concentrations of the two glucuronide metabolites, M6G and M3G and would provide and maintain comparable anti-nociception to IV morphine with less adverse effects, following single and multiple doses. Thermal nociception tests are commonly used in experimental studies of the anti-nociceptive effects of opioids (21), therefore, a thermal noxious stimulus was chosen for threshold testing in the current study, with the temperature at which a response was observed being deemed the nociceptive threshold.

## 2 Materials and methods

### 2.1 Animals

Six healthy, University-owned, Thoroughbred horses (age: 4–7 years; weight: 543 ± 65.7 kg) were studied. The number of horses selected for this study was based on a power analysis using the thermal threshold as the end point. Based on data from a previously published study (16), a sample size of 4 horses was determined sufficient to detect a 2°C difference with a standard deviation of 1.4°C, a power of 0.9 and alpha of 0.05. An additional two horses were added to allow for some variation in observed values from hypothetical values used for sample size calculations, giving a sample size of six total horses. Horses did not receive any medications for a minimum of two weeks prior to commencement of the study. Before beginning the study, horses were determined healthy by physical examination, complete blood count (CBC) and a serum biochemistry panel. Blood samples were submitted to the Clinical Pathology Laboratory of the William R. Pritchard Veterinary Medical Teaching Hospital of the University of California, Davis, for analyses. Horses were moved to 12 × 12 foot stalls a minimum of 72 h prior to drug administration. The study was conducted in accordance with the Institutional Animal Care and Use Committee of the University of California at Davis (#23392).

### 2.2 Instrumentation and drug administration

**Figure 1:**
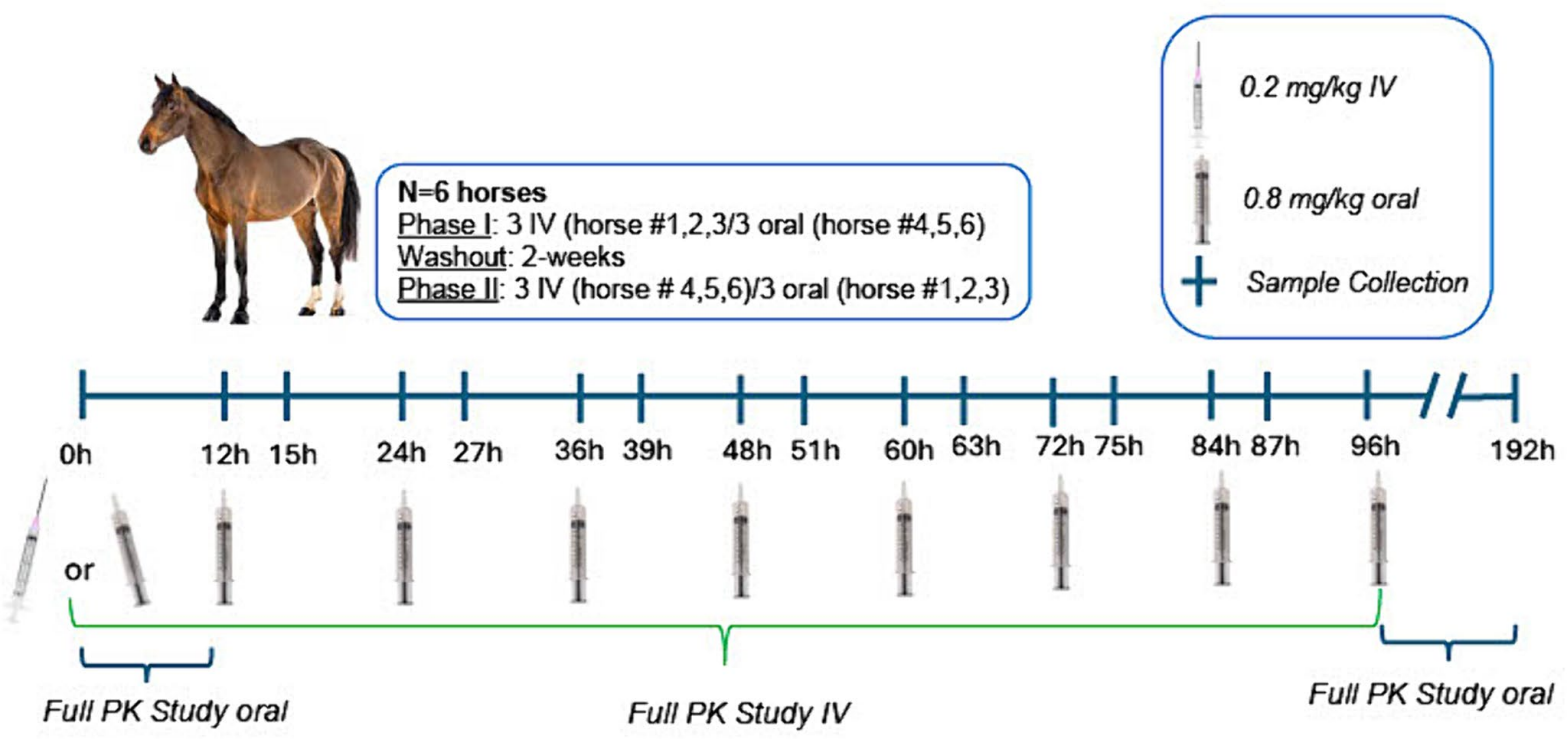
Study design timeline, including dosing and sample collection protocol for intravenous (0.2 mg/kg single administration) and oral administration (0.8 mg/kg q12h × 9 doses) of morphine. For full pharmacokinetic studies, samples were collected at 0, 5, 10, 15, 30, and 45 min and 1, 2, 3, 4, 5, 6, 8, and 12, 18, 24, 30, 36, 48, 72, and 96 h post IV administration and at 0, 5, 10, 15, 30, and 45 min and 1, 2, 3, 4, 5, 6, 8, and 12 h following the first oral dose and 5, 10, 15, 30, and 45 min and 1, 2, 3, 4, 5, 6, 8, and 12, 18, 24, 30, 36, 48, 72, and 96 h following the final oral dose (administered at 96 h). Two venous catheters were placed prior to IV drug administration (one for drug administration and one for sample collection). One venous catheter was placed prior to the first oral dose and again prior to the last oral dose for sample collection. Dosing catheters (IV administration) were removed after drug administration. Sampling catheters were removed following the 18 h sample collection and additional samples collected by direct venipuncture. Behavioral observations were made at each blood sample collection time point, prior to collection of the blood sample.

This study was conducted in a balanced, randomized 2-way cross-over design, whereby 3 horses received IV morphine and 3 oral morphine in Phase 1 and Phase II (Figure 1). The order of treatment (IV versus oral) for each horse was determined using a computerized random number generator.^1^ For IV administration, horses received a single oral dose of 0.2 mg/kg morphine and for oral administration, multiple oral doses of 0.8 mg/kg of morphine sulfate. A minimum 2 week washout period, after the final dose, was observed between treatments for each horse. An oral dose of 0.8 mg/kg was selected based on a previously conducted study, whereby M6G concentrations exceeded those achieved following IV administration of a therapeutic dose with less adverse effects (6). For oral administration, horses received a dose every 12 h for 4.5 days (total of 9 doses). This duration of administration was selected based on a previously published study in humans describing a 2-fold accumulation of the active metabolite at the effector site along with a 4-fold higher potency compared to a single dose (19). Morphine sulfate tablets were suspended in water (10 mL) and delivered via a dosing syringe directly into the oral cavity. Horses were fed approximately 3 h prior to dosing. Any residual food was removed from the oral cavity prior to drug administration.

Prior to drug administration on day 1 and oral administration on day 5, a 14-gauge catheter was placed, in one external jugular vein for sample collection. Sampling catheters were removed at 18 h post administration and subsequent samples collected via venipuncture. Horses receiving IV morphine had a second catheter placed (in the contralateral jugular vein) for drug administration. The dosing catheter was removed following dosing.

### 2.3 Sample collection

Following IV administration, blood samples (10 mL/time point; total of 210 mL) for drug concentration determination were collected at time 0 (prior to drug administration) and at 5, 10, 15, 30, and 45 min, and 1, 2, 3, 4, 5, 6, 8 and 12, 18, 24, 30, 36, 48, 72 and 96 h. Following administration of the first oral dose, blood samples (10 mL/time point; total of 490 mL) were collected at time 0, 5, 10, 15, 30, and 45 min and 1, 2, 3, 4, 5, 6, 8, and 12 h. Additional blood samples were collected every 12 h, immediately prior to drug administration (trough) and at 3 h post morphine administration [*C*_max_ for morphine + M6G (6)] in the oral dosing group. After the final oral dose, blood samples were collected at 5, 10, 15, 30, and 45 min, and 1, 2, 3, 4, 5, 6, 8 and 12, 18, 24, 30, 36, 48, 72, and 96 h. Blood samples were collected into EDTA blood tubes, placed on ice and centrifuged at 3,000 × g. Plasma was immediately transferred into storage cryovials and stored at −20°C (maximum of 4 weeks) until analyzed for drug concentration determination.

### 2.4 Concentration determination and pharmacokinetic analysis

Morphine, morphine-3-glucuronide (M3G) and M6G concentrations were determined using previously published and validated liquid chromatography–tandem mass spectrometry methods (1, 2). Details of the analytical method can be found in the Supplementary section. Pharmacokinetic analysis was conducted using a commercially available pharmacokinetic software program (Phoenix WinNonlin v8.2, Certara, Princeton, NJ). Initial estimates of pharmacokinetic parameters for subsequent model fitting were determined by non-compartmental analysis (NCA) using a commercially available computer software program (Phoenix WinNonlin v8.3, Certara, Princeton, NJ). Non-compartmental analysis was also used for determination of steady state pharmacokinetics following oral administration.

Following NCA, concentration data for all horses following both routes of administration were modeled simultaneously using a nonlinear mixed effects compartmental modeling (NLME) approach with the Phoenix NLME software program. As part of the model building process, the first-order conditional estimation method with interaction (FOCE-ELS) was used and two and three compartment models with saturable and linear absorption were evaluated. Residual error models assessed included additive, multiplicative, Poisson, and mixed additive/multiplicative residual error models. Random effects were included for all structural parameters and were modeled with log linear functions. Visual analysis of the observed versus predicted concentration graphs, residual plots, Akaike Information Criterion and %CV of parameter estimates were all considered in assessing which model provided the best fit.

Pharmacokinetic analysis was conducted on M6G and M3G concentrations using NCA as described for determination of initial estimates for morphine. The metabolic ratios were calculated at each timepoint using the formula:


$$\mathrm{Metabolic}\;\mathrm{ratio}=\left({\mathrm{Concentration}}_{\mathrm{metabolite}}/{\mathrm{Concentration}}_{\mathrm{morphine}}\right)\text{.}$$

### 2.5 Physiological responses and behavioral monitoring

Behavioral observations were conducted prior to entering the stall for collection of blood samples at each time point. Gastrointestinal borborygmi was recorded prior to and at 30 and 45 min and 1, 2, 4, 6, 8 and 24 h following the first and last dose. Auscultation was performed for 15 s at each abdominal quadrant and borborygmi recorded; a numerical score equal to the number of borborygmi per 30 s in each quadrant was assigned. Defecation incidence as well as fecal consistency were monitored throughout the sampling period. Observers were not blinded to treatment.

### 2.6 Assessment of thermal nociception

A commercially available wireless device (Topcat Metrology, United Kingdom) was used for thermal nociceptive testing. An area on the outside of the metacarpus was shaved three days prior to commencement of the study. For measurements, the temperature probe was placed in direct contact with the skin and the nylon strap tightened around the leg. The device was then be activated (heat applied) by use of a wireless hand-held toggle switch located outside the stall. Once a response was noted (i.e., kicking, pawing, abruptly lifting the leg, touching leg with nose, etc.) heat was discontinued. If no response was noted, the device automatically turned off when the probe reaches 55°C (automatic shut-off to avoid burns).

For intravenous administration (day 1), a baseline measurement was recorded (30 min to 1 h prior to treatment) and additional measurements taken at 15, 30, 45, 60, 90, 120, 180, 240 and 360 min post treatment. For oral administration, on the first day of drug administration, (day 1) a baseline measurement was taken (30 min to 1 h prior to treatment) and additional assessments made at 15, 30, 45, 60, 90, 120, 180, 240 and 360 min post treatment. On days 2, 3, and 4, measurements were taken prior to and at 3 h post morning drug administration. On day 5, following the morning treatment (final treatment), a baseline measurement was recorded, and additional measurements taken at 15, 30, 45, 60, 90, 120, 180, 240, and 360 min post treatment.

### 2.7 Statistical analysis

Thermal nociceptive thresholds were recorded and standardized to thermal excursion (%TE) for comparability, as described previously (22), using the formula: %TE = 100 × [(T_T_ − T_0_)/(T_C_ − T_0_)] where T_T_ represents the thermal threshold, T_0_ the skin temperature and T_C_ the thermal nociception cut-off temperature.

Statistical analyses were conducted using commercially available software (Stata/BE 17.0, StataCorp, TX, United States) to assess significant differences in thermal threshold, thermal excursion and gastrointestinal borborygmi scorese between baseline and each time point for IV and oral administration. Gastrointestinal borborygmi scores following the last dose were also compared to pre administration values for both the first and last dose. All analyses were done with mixed effects analysis of variance, in which the horse was the random effect, and time the fixed effect. *Post hoc* comparisons were performed using a Bonferroni multiple comparison adjustment to preserve a nominal significance level of *p* < 0.05.

## 3 Results

All horses remained healthy throughout the dosing periods and were able to complete the study. Dosing and sample collection were uneventful.

The responses for all analytes were linear and gave correlation coefficients of 0.99 or better. Quality control samples (*n* = 6) for the analytes were assayed in replicates for determination of precision and accuracy (*n* = 6). Accuracy was reported as percent nominal concentration and precision as percent relative standard deviation. Accuracy and precision were all within ±10% (Supplementary Table S1). The limit of quantitation (LOQ) was 0.25 ng/mL for morphine and 0.1 ng/mL for M6G and M3G and the limit of detection (LOD) was 0.1 ng/mL for morphine and 0.05 ng/mL for M3G and M6G.

**Figure 2:**
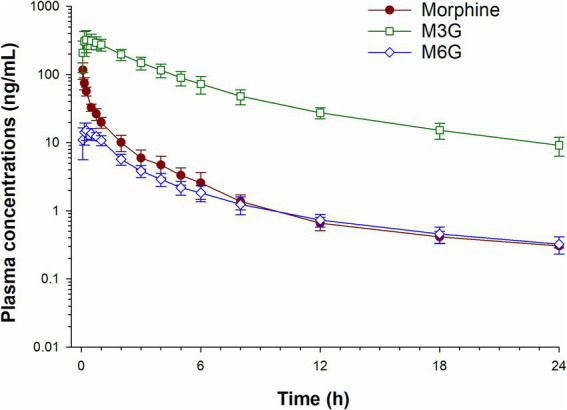
Plasma concentration time curves for morphine, morphine 3-glucuronide (M3G) and morphine 6-glucuronide (M6G) following a single intravenous administration of 0.2 mg/kg.

**Figure 3:**
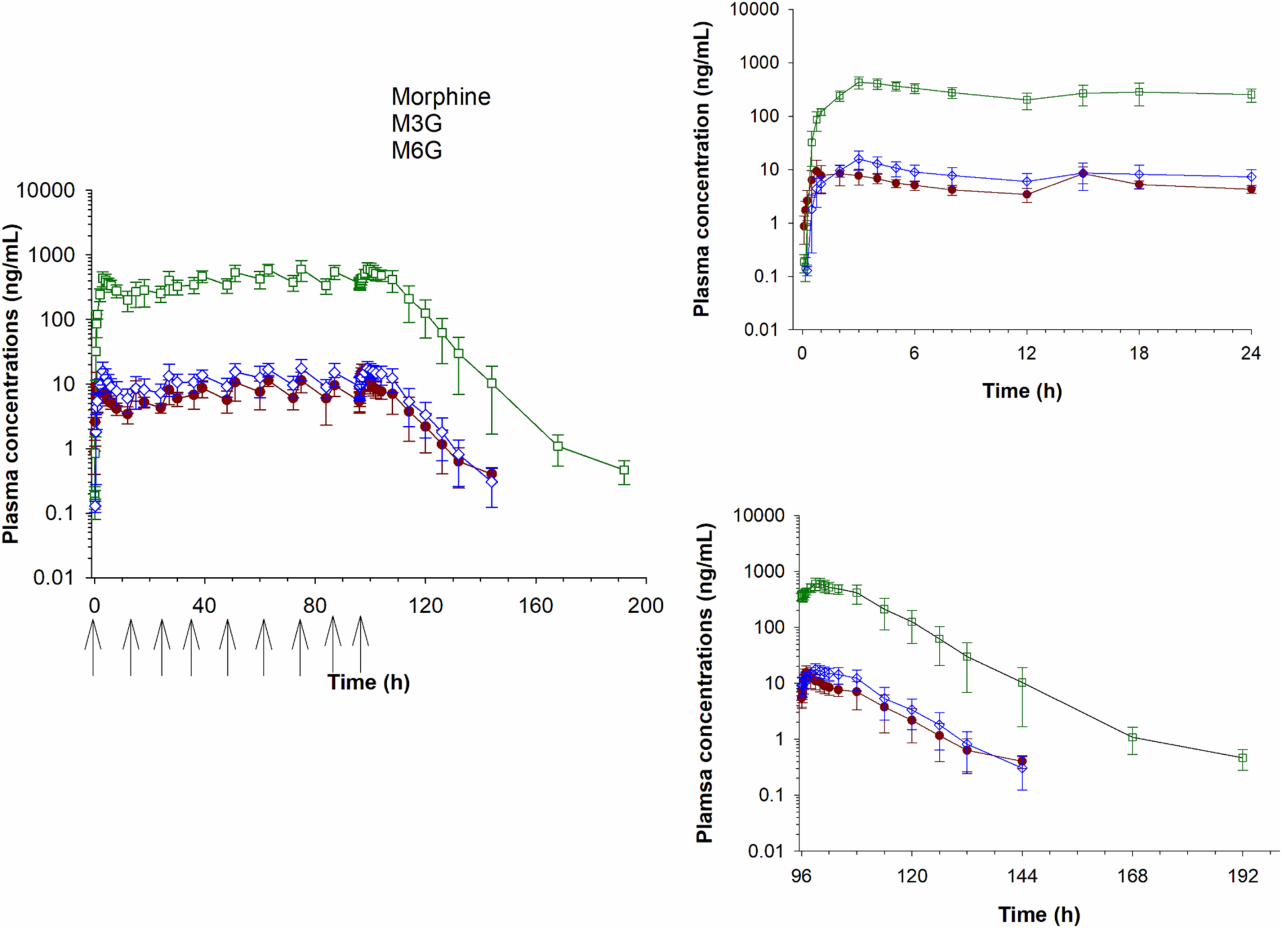
Plasma concentration time curves for morphine, morphine 3-glucuronide (M3G) and morphine 6-glucuronide (M6G) following oral administration of 0.8 mg/kg q12 hours for a total of 9 doses. Inset graphs show the concentrations after the first and last dose. Arrows represent dosing times.

Plasma concentrations of morphine, M6G and M3G following a single IV administration (0.2 mg/kg) are depicted in Figure 2 and concentrations following oral administration (0.8 mg/kg) in Figure 3. For both oral and IV administration, the predominant metabolite was M3G. Although *T*_max_ differed, the maximum M6G concentrations were similar between routes of administration. The maximum M6G concentration following the first oral dose occurred at 3 h.

Select pharmacokinetic parameters for morphine following NCA are listed in Table 1. Following oral administration, *C*_max_ following the last dose was slightly higher (15.7 ± 4.24 ng/mL; average ± SD) than after the first dose (11.8 ± 4.30 ng/mL), resulting in an accumulation index of 1.51 ± 0.14. The time of maximum concentration (*T*_max_) was similar for the first and last dose (Table 1). The final pharmacokinetic model was a 3-compartment population model, parameterized with respect to clearance. A multiplicative residual error model was used. Diagnostic plots for the final NLME pharmacokinetic model used are provided as Supplementary Figures S1, S2. Pharmacokinetic parameters (estimate and coefficient of variation) for the joint fitting of intravenous and oral data are listed in Table 2.

**Table 1:** Select pharmacokinetic parameters for morphine generated from non-compartmental analysis following a single intravenous administration of 0.2 mg/kg and multiple oral administrations (0.8 mg/kg q12 hours for a total of 9 doses) to six horses.

Parameter	Intravenous		Oral	
	Mean ± SD	Median (Range)	Mean ± SD	Median (Range)
C(0) (ng/mL)	184.0 ± 63.5	177.8 (98.1–271.2)	NA	NA
*C*_max_ (ng/mL)				
After first dose	–	–	11.8 ± 4.30	11.1 (7.12–18.0)
At steady state	–	–	15.7 ± 4.24	15.4 (10.4–21.8)
*T*_max_ (h)				
After first dose	–	–	2.15 ± 1.05	2.5 (0.75–3.0)
At steady state	–	–	98.7 ± 4.51	97.0 (96.75–108)
*C*_min_ (ng/mL)				
After first dose	–	–	3.30 ± 1.00	3.45 (2.09–4.66)
At steady state	–	–	4.86 ± 1.86	4.70 (3.17–7.92)
AUC_inf_ (ng hr./mL)	100.4 ± 14.0	101.0 (81.1–114.1)	176.6 ± 58.0	172.6 (120.2–261.6)
AUC_tau_ (ng hr./mL)	–	–	108.0 ± 27.1	112.4 (73.8–151.6)
*C*_avg_ (ng/mL)	–	–	9.00 ± 2.26	9.37 (6.14–12.6)
Terminal HL (h)	8.06 ± 3.20	9.72 (3.92–13.6)	7.53 ± 1.25	7.59 (6.26–9.84)
Fluctuation (%)	–	–	117.4 ± 26.0	122.3 (76.1–146.6)
Accumulation Index	–	–	1.51 ± 0.14	1.50 (1.36–1.75)

Unless otherwise stated, values reported for oral administration represent parameters at steady state. –, not applicable; *C*_max_, maximum plasma concentration; *T*_max_, time of maximum plasma concentration; *C*_min_, minimum plasma concentration; AUC, area under the concentration time curve; Terminal HL, terminal half-life.

**Table 2:** Model typical values (tv) for morphine following a single intravenous (0.2 mg/kg) and multiple oral (0.8 mg/kg q12 hours for 9 doses) administration to horses (*n* = 6).

Parameter	Estimate	CV (%)
*Fixed effects*		
tvKa (1/h)	0.101	5.49
tvV (L/kg)	1.72	20.6
tvV2 (L/kg)	6.47	22.9
tvV3 (L/kg)	1.87	18.4
tvCl (mL/min/kg)	35.4	6.24
tvCl2 (mL/min/kg)	7.31	16.5
tvCl3 (mL/min/kg)	18.7	19.7
tvFbio (%)	35.9	8.35
stdev0	0.016	4.30
*Random effects: between subject variability (% CV)*		
Ka	2.06 × 10^−7^	0.06
V	0.203	47.4
V2	0.007	8.20
V3	1.47 × 10^−5^	0.38
Cl	0.012	10.8
Cl2	9.66 × 10^−8^	0.03
Cl3	0.011	10.7

tvKa, rate of absorption; tvV, the volume of the central compartment; tvV2, denotes the volume of the peripheral compartment; tvV3, denotes the volume of the deep peripheral compartment; tvCl, the clearance of drug from serum; tvCL2, the clearance of drug from the peripheral compartment; tvCL3, the clearance of drug from the deep peripheral compartment; tvFbio, bioavailability; stdev0 = the estimated residual standard deviation for serum data.

Table 3 lists the pharmacokinetic parameters for M6G following IV and oral administration. For M6G, *C*_max_ (mean ± SD) was 14.9 ± 4.27 ng/mL following IV administration and 15.9 ± 6.18 ng/mL (after the first dose) and 18.9 ± 3.50 ng/mL (after the last dose) following oral administration. The *T*_max_ (median) was 0.25 and 3.0 h for IV and oral (after the first and last dose) administration, respectively. The maximum concentration (mean ± SD) for M3G was 327.8 ± 105.2 ng/mL following IV administration and 440.8 ± 104.8 ng/mL (first dose) and 643.6 ± 128.7 ng/mL (last dose) after oral administration (Table 4). The median *T*_max_ was 0.38 h following IV administration and following oral administration, 3.0 h and 4 h after the first and last dose, respectively (Table 4). The accumulation index (mean ± SD) for M6G and M3G was 1.35 ± 0.089 and 1.58 ± 0.126, respectively (Tables 3, 4).

**Table 3:** Pharmacokinetic parameters for morphine 6-glucuronide (M6G) following a single intravenous administration of 0.2 mg/kg to and oral administration of 0.8 mg/kg q12 hours for nine doses to six adult horses.

	Intravenous		Oral	
	Mean ± SD	Median (Range)	Mean ± SD	Median (Range)
*C*_max_ (ng/mL)				
After first dose	14.9 ± 4.27	15.2 (10.9–22.9)	15.9 ± 6.18	14.2 (11.9–28.3)
At steady state			18.9 ± 3.50	20.4 (13.3–23.0)
*T*_max_ (h)				
After first dose	0.259 ± 0.223	0.25 (0.16–0.75)	3.0 ± 0	3.0 (3.0–3.0)
At steady state	–	–	101.7 ± 4.79	99.0 (99–108)
*C*_min_ (ng/mL)				
After first dose	–	–	5.61 ± 3.46	5.46 (3.08–10.1)
At steady state	–	–	8.41 ± 3.42	7.79 (5.66–15.0)
AUC_inf_ (h*ng/mL)	50.4 ± 7.53	53.4 (39.0–59.4)	267.6 ± 80.5	256.3(178.8–387.7)
AUC_tau_ (ng hr./mL)	–	–	167.6 ± 38.4	167.4 (132.0–232.8)
*C*_avg_ (ng/mL)	–	–	14.0 ± 3.21	13.9 (11.0–19.4)
Terminal HL (h)^†^	9.59 ± 2.64	9.71 (6.67–14.3)	6.05 ± 0.869	6.12 (4.71–7.25)
Fluctuation (%)	–	–	72.3 ± 20.2	73.9 (41.6–96.1)
Accumulation Index	–	–	1.35 ± 0.089	1.35 (1.20–1.46)

Parameters were generated using noncompartmental analysis. –, not applicable; *C*_max_ is the maximum measured plasma concentration, *t*_max_ is the time of the maximum plasma concentration, *C*_min_, minimum plasma concentration, AUC_inf_ is the area under the curve extrapolated to infinity, *λ*_Z_ is the slope of the terminal elimination curve, half-life *λ*_Z_ is the terminal half-life.

**Table 4:** Pharmacokinetic parameters (geometric mean or median (*T*_max_) and range) for morphine 3-glucuronide (M3G) following a single intravenous administration of 0.2 mg/kg to and oral administration of 0.8 mg/kg q12 hours for nine doses to six adult horses.

	Intravenous		Oral	
	Mean ± SD	Median (Range)	Mean ± SD	Median (Range)
*C*_max_ (ng/mL)				
After first dose	327.8 ± 105.2	318.6 (250.6–540.9)	440.8 ± 104.8	424.9 (284.9–596.1)
After last dose	–	–	643.6 ± 128.7	672.6 (499.4–857.2)
*T*_max_ (h)				
After first dose	0.397 ± 0.292	0.375 (0.25–1.0)	3.33 ± 0.52	3.00 (3.00–4.00)
After last dose	–	–	102.3 ± 4.46	100.0 (99.0–108.0)
*C*_min_ (ng/mL)				
After first dose	–	–	191.4 ± 70.2	216.4 (116.3–261.1)
At steady state	–	–	318.1 ± 41.3	320.5 (262.0–379.2)
AUC_inf_ (h*ng/mL)	1541.2 ± 266.2	1572.9 (1261.8–1862.2)	9688.1 ± 2144.8	9425.4 (7250.7–13717.8)
AUC_tau_ (ng hr./mL)	–		5883.9 ± 749.4	5601.1 (5289.5–7201.1)
*C*_avg_ (ng/mL)	–		490.3 ± 62.4	466.8 (440.8–600.1)
Terminal HL (h)^†^	9.85 ± 3.47	9.18 (7.56–16.7)	8.19 ± 1.12	7.87 (7.28–10.4)
Fluctuation (%)	–		63.8 ± 19.8	68.2 (43.5–89.8)
Accumulation Index	–		1.58 ± 0.126	1.53 (1.47–1.81)

Parameters were generated using noncompartmental analysis. –, not applicable; *C*_max_ is the maximum measured plasma concentration, *t*_max_ is the time of the maximum plasma concentration, *C*_min_, minimum plasma concentration AUC_inf_ is the area under the curve extrapolated to infinity, *λ*_Z_ is the slope of the terminal elimination curve, half-life *λ*_Z_ is the terminal half-life.

**Figure 4:**
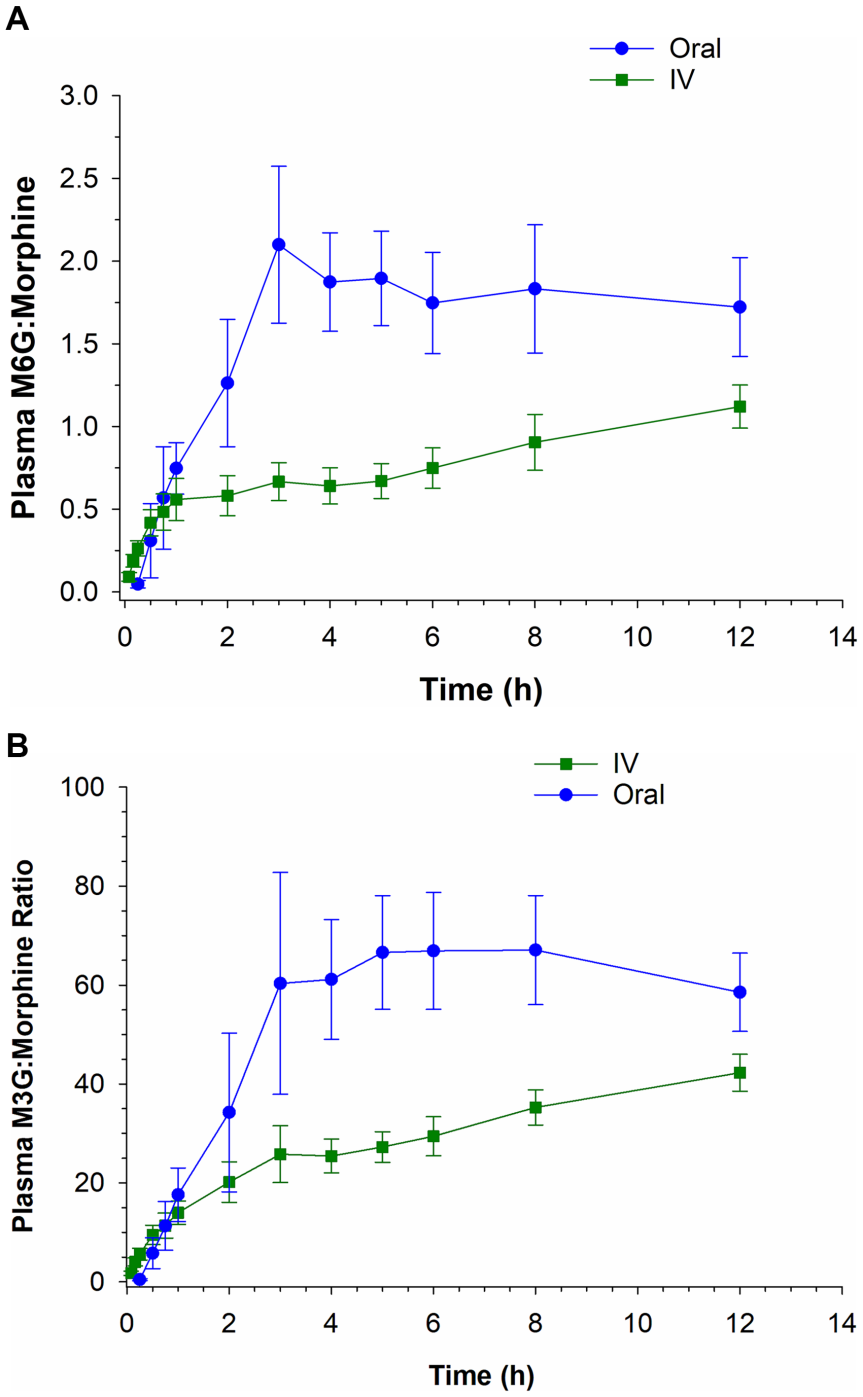
Ratio of morphine 6-glucuronide (M6G) to morphine **(A)** and morphine 3-glucuronide (M3G) to morphine **(B)** following a single intravenous (IV) administration of 0.2 mg/kg and oral administration of 0.8 mg/kg.

The plasma M6G:morphine and M3G:morphine ratios following oral and IV administration over the first 12 h post administration are depicted in Figures 4A,B. For M6G, the ratio of metabolite to morphine was greater following IV administration compared to oral for the first 15 min post administration and higher for oral administration thereafter. In the case of M3G, the ratio of metabolite to parent was greater for IV for the first 45 min post administration and greater for oral administration for the remaining time points (see Figures 4A,B).

**Figure 5:**
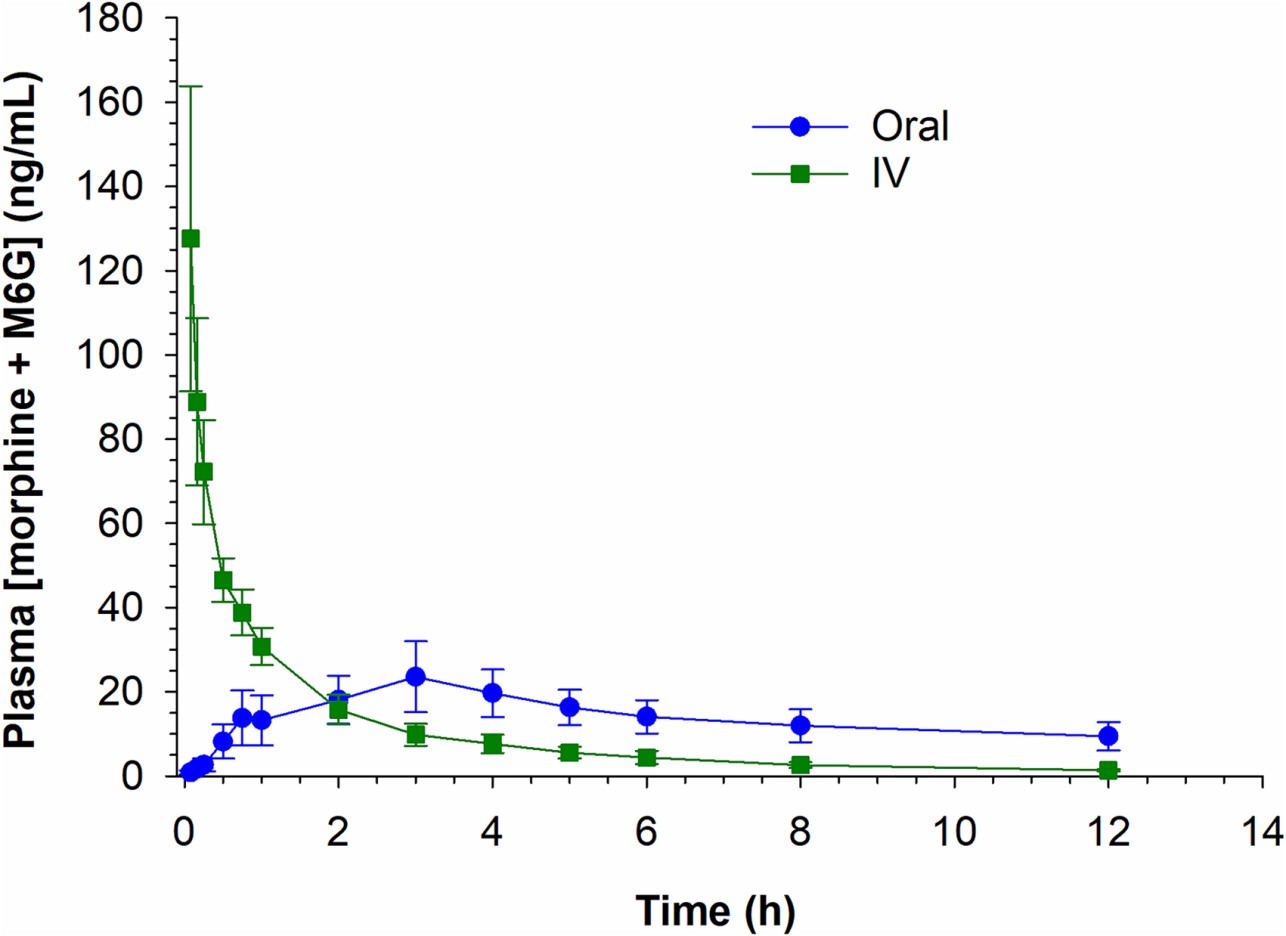
Combined morphine 6-glucuronide (M6G) and morphine concentrations following a single intravenous (IV) administration of 0.2 mg/kg and oral administration of 0.8 mg/kg.

Plasma concentrations of morphine and M6G (the two anti-nociceptive compounds) for both routes of administration were summed and are depicted in Figure 5. The combined plasma concentrations (morphine + M6G) were equivalent in the IV and oral groups by 2 h post administration with concentrations remaining higher in the oral group at all subsequent sampling times.

Immediately following IV administration, 4/6 horses demonstrated signs of agitation including headshaking, pawing, and circling in the stall. These signs resolved in all horses by 30 min post administration. One horse developed hives at 30 min post IV administration, with resolution occurring by 4 h. Oral administration was well tolerated in 5/6 horses with one horse demonstrating signs of mild colic (pawing) at 12 h post administration of the first dose (immediately prior to administration of the second dose). The horse appeared more comfortable by the next administration and did not exhibit discomfort for the remained of the treatment period. Signs of agitation (pawing, headshaking, and circling) were not observed following oral administration.

Following IV administration, gastrointestinal borborygmi scores were significantly decreased (*p* < 0.05), relative to pre-treatment, until 6 h post administration (Table 5). Following oral administration, significant decreases in gastrointestinal borborygmi scores, relative to baseline, were noted at the 30 and 45 min and 1 and 2 h assessments (Table 5). There were no significant differences in scores noted at any times following the final dose, compared to the 96 h administration (pre-final dose).

**Table 5:** Gastrointestinal scores (mean ± SD), following a single intravenous administration of 0.2 mg/kg and oral administration of 0.8 mg/kg q12 hours for 9 doses to six adult horses.

Time (h)	0.2 mg/kg IV	0.8 mg/kg PO
Baseline	3.0 ± 0.5	2.4 ± 1.1
0.5	0.4 ± 0.4^*^	0.9 ± 0.7^*^
0.75	0.8 ± 0.7^*^	1.2 ± 0.7^*^
1	1.1 ± 0.4^*^	1.3 ± 0.9^*^
2	0.7 ± 0.8^*^	0.8 ± 0.9^*^
4	1.8 ± 0.9^*^	2.2 ± 0.6
6	1.7 ± 0.7^*^	2.1 ± 0.9
8	2.1 ± 0.5^*^	2.0 ± 0.3
24	2.8 ± 0.8	1.9 ± 0.9
96	–	2.2 ± 0.9
96.5	–	1.6 ± 1.2
96.75	–	1.6 ± 1.2
97	–	1.6 ± 0.9
98	–	1.6 ± 1.1
100	–	2.2 ± 0.9
102	–	1.9 ± 0.9
104	–	2.5 ± 1.0
120	–	2.4 ± 0.9

–, not applicable. *, significantly (*p* < 0.05) different from baseline (prior to first dose).

**Figure 6:**
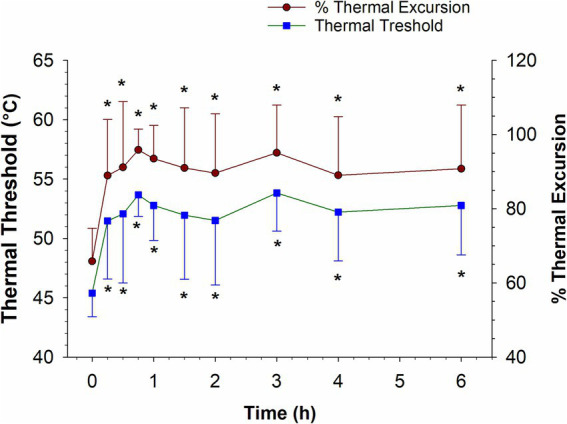
Thermal threshold and thermal excursion following a single intravenous administration of 0.2 mg/kg morphine to six horses. *, indicates a significant (*p* < 0.05) difference from baseline.

**Figure 7:**
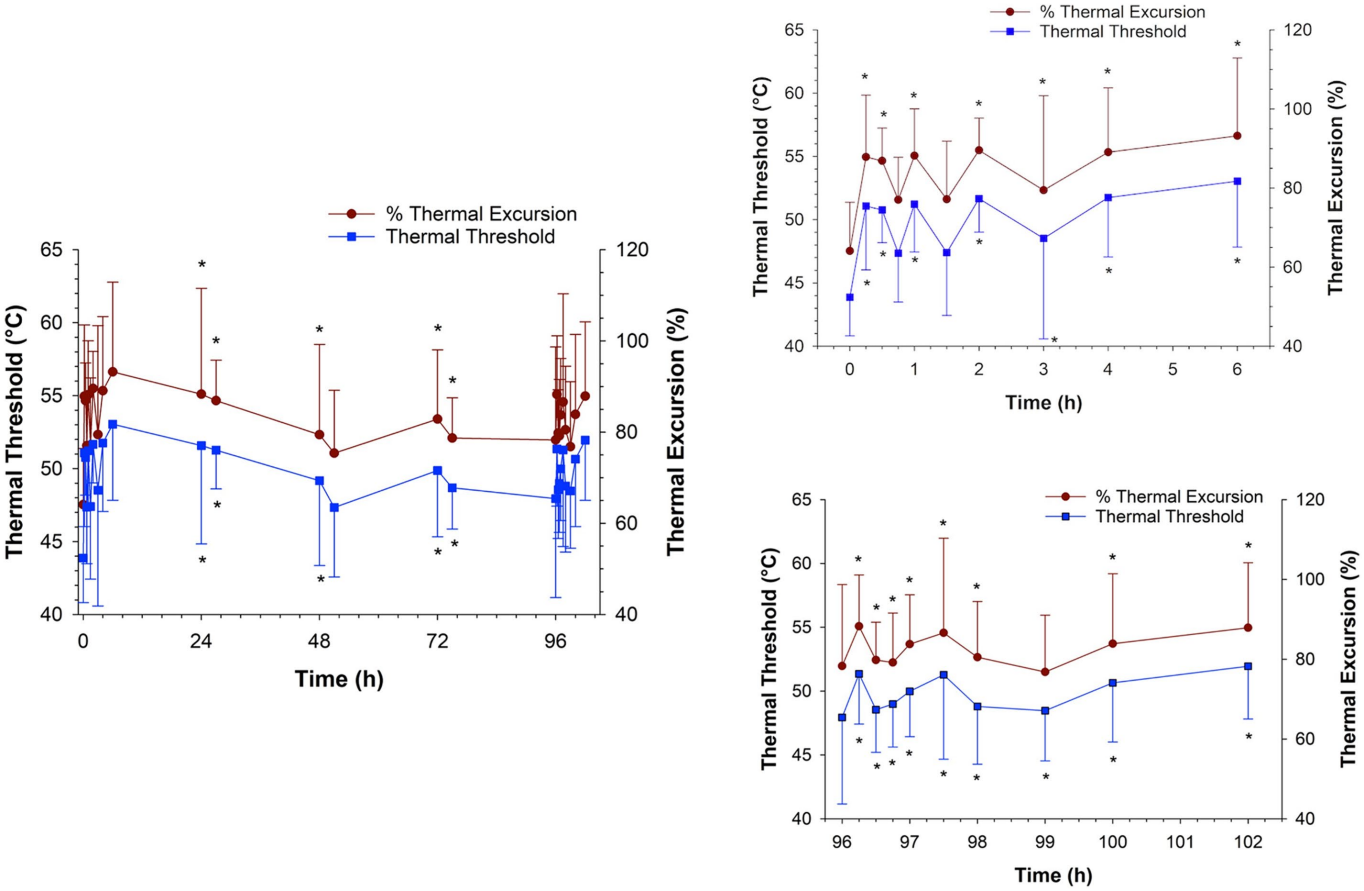
Thermal threshold and thermal excursion following oral administration of 0.8 mg/kg q12 hours of morphine for a total of 9 doses to six horses. Inset graphs show the values after the first and last dose. *, indicates a significant (*p* < 0.05) difference from baseline (pre administration of first dose).

The thermal threshold and thermal excursion were significantly increased (*p* < 0.05), relative to baseline, at all times post administration following IV administration (Figure 6). The effects of oral morphine on thermal nociception are depicted in Figure 7. Following oral administration, the thermal threshold and thermal excursion were significantly increased (*p* < 0.05), relative to baseline, at all times with the exception of 45 min and 1.5 and 96 h post administration (Figure 7).

## 4 Discussion

The pharmacokinetics of a single administration of oral morphine in horses has been described previously (6). The current study provides additional pharmacokinetic data as well as providing information regarding the thermal anti-nociceptive effect of morphine after a single and multiple oral administrations. Plasma concentrations following oral administration in the current study agreed with the previous report (6). Maximum concentrations (*C*_max_) ranged from 7.12–18.0 ng/mL and 10.4–21.8 ng/mL for the first and last dose, respectively, similar to that reported by Poth and colleagues following a single dose (6.19–18.9 ng/mL) (6). The oral bioavailability in the two studies were also similar (35.9 and 33.5%) (6).

Since morphine is classified as a high-extraction ratio drug and highly susceptible to the first pass-effect, it was theorized that oral administration would result in higher concentrations of the two glucuronide metabolites, M6G and M3G. In the case of morphine, susceptibility to the first pass effect may be beneficial, given the anti-nociceptive effects of the M6G metabolite (11–16). As expected, the ratios of the metabolites to the parent compound (M6G:morphine and M3G:morphine) were generally higher following oral administration compared to IV. Additionally, as reported previously (6) the M6G *C*_max_ following oral administration of 0.8 mg/kg was comparable that following IV administration of 0.2 mg/kg. Since the analgesic effects of morphine have been attributed to both morphine and the M6G metabolite, the combined plasma concentration of the two compounds was also assessed at each time point. The sum of the morphine and M6G concentrations were higher following IV administration, compared to oral, for the first 2 h following drug administration, attributable to the higher concentrations of the parent compound. After two hours the combined plasma concentration (morphine and M6G) following oral administration exceeded that observed following IV administration.

In addition to non-compartmental analysis, a compartmental population pharmacokinetic model was used to analyze morphine concentration data. A 3-compartment model with joint fitting of IV and oral routes gave the best fit to plasma concentration data. A 3-compartment model has been used in previous studies describing the pharmacokinetics of IV morphine in horses (1, 23). In the study conducted by Poth and colleagues, a 2-compartment joint IV and oral pharmacokinetic model best fit morphine concentration data following a single administration (6). The reason for the discrepancy in the number of compartments between the previous study and the current one is not immediately clear but may be due to differences in the drug administration protocols (multiple versus single administration) and different sampling time points. The study design of the current study may have allowed for identification of a third compartment.

The terminal half-life for both routes of administration was similar to previous reports (1, 2, 6). As has been suggested for humans (19), following multiple oral administrations of morphine at 12 h intervals, there was only minimal accumulation of both morphine (accumulation index of 1.35) and M6G in the plasma. Using pharmacokinetic modeling, Lotsch and colleagues suggested that although M6G does not accumulate in plasma with a 12 h dosing interval, that this dosing regimen may allow for accumulation at the target site, thus leading to a greater analgesic effect then would be observed with a single dose (19).

As reported previously (16, 24), in the current study, IV administration of 0.2 mg/kg morphine resulted in a thermal anti-nociceptive effect lasting at least 6 h (the last time point assessed). The lowest morphine concentration coinciding with thermal anti-nociception was 2.57 ng/mL (mean) and the lowest combined morphine, M6G concentration was 4.41 ng/mL. It is important to recognize that 6 h was the last time point assessed and therefore the thermal anti-nociceptive effect may continue beyond 6 h, therefore the minimum effective concentration may be lower than the 6 h concentrations.

As with IV administration, a significant thermal anti-nociceptive effect was observed starting as early as 15 min following oral administration. The corresponding mean plasma concentrations of morphine and the combined morphine and M6G concentrations were 2.60 and 2.67 ng/mL, respectively. Additional assessments of effects on thermal nociception were made at 24 h post administration (mean morphine concentration of 4.29 ng/mL and morphine + M6G concentration of 11.6 ng/mL) through the last dose (96 h). Starting with the second dose (12 h post administration of the first dose) until the final dose, effects on thermal nociception were assessed immediately prior to drug administration (trough) and at 3 h post administration (peak). Three hours was chosen based on previous studies, where the combined morphine, M6G concentration was maximal at this time (6). Apart from the 51- and 96 h assessments, significant thermal anti-nociceptive effects were noted at all times during the treatment period. After the final dose, a significant thermal anti-nociceptive effect, compared to the baseline value (prior to the first dose), was observed starting at 15 min post administration. This effect continued through at least 6 h post administration (the final time point assessed). Previous reports in humans, demonstrated that oral morphine is more effective following multiple doses (18). As described previously, one group of investigators suggested that this finding, at least in part, was due to accumulation of M6G at the effector site (19). In contrast, in the current study, while the results do indicate a sustained thermal anti-nociceptive effect with repeated administration, the effect does not appear to be greater with multiple administrations compared to a single oral administration.

As reported previously, IV administration of morphine resulted in signs of agitation (headshaking, pawing, circling in the stall) immediately upon administration (1–3, 6, 10). Also as has been reported with morphine administration in horses and other species, one horse in the IV group developed hives, presumably due to histamine release associated with drug administration (25–27). Slow circling in the stall was noted for one horse in the oral dosing group. In a previous study, whereby horses received a single oral dose of 0.8 mg/kg, responses were somewhat variable with some horses standing quietly and others slowly circling in their stalls for the first 30 min post administration (6). The irregularity in responses is not unexpected as individual variability with respect to behavioral responses is known to occur following opioid administration to horses.

Morphine administration has been associated with decreased gastrointestinal borborygmi scores (2, 5, 6, 10). In the current study, a significant decrease in gastrointestinal borborygmi scores was observed until 6 h post administration following IV administration. Although notably, there were no assessments between 6 and 24 h, gastrointestinal borborygmi scores were not significantly different from baseline at 24 h post administration. Following oral administration, significant decreases in gastrointestinal borborygmi scores were noted from 30 min to 2 h post administration of the first dose. Poth and colleagues reported similar findings following a single oral administration of morphine at doses of 0.2, 0.6, and 0.8 mg/kg (6). Significant differences in gastrointestinal borborygmi scores were not noted after 2 h or following the final dose when compared to pre-administration scores for the first (baseline) or final dose (96 h).

While results of the current study are encouraging for the use of oral morphine in horses there are notable limitations to this study and additional studies are necessary before this drug can be recommended for clinical use. One important consideration is the number and population of horses studied. Although the number of animals studied was sufficient to detect significance, the sample size was still small, and it would be prudent to study oral morphine in a larger population of horses. It has been well established that a large degree of variability exists in the pharmacokinetics of morphine in horses, which could impact therapeutic efficacy and the likelihood of adverse effects (6). It should also be noted that the horses studied here were healthy research horses and results may differ in horses experiencing pain. Additionally, while the thermal nociceptive model used in the current study has been well described and is useful for controlled assessments, it is an experimental model and may not be representative of all clinical scenarios, such as pain originating from an inflammatory response. As mentioned above, effects on thermal nociception were only assessed for 6 h following the final oral dose and because a significant anti-nociceptive effect was observed at that final assessment, it is not possible to determine the minimum effective concentration. Additional assessments at later times post administration would be useful to determine the minimum effective concentration and conduct pharmacokinetic-pharmacodynamic modeling. It is also important to note that the observers were not blinded to the treatments each horse received when recording behavioral assessments, which could result in bias.

As hypothesized, oral administration of 0.8 mg/kg morphine provided and maintained comparable anti-nociception effects to IV morphine with less adverse effects, following single and multiple doses. Morphine was well tolerated following oral administration with less excitation and minimal effects on gastrointestinal borborygmi scores compared to IV administration. Results of the current study are encouraging for study of the anti-nociceptive effects and safety of oral morphine administration to horses. With respect to safety, since a limited number of horses were studied and one horse did develop clinical signs of colic post oral administration, additional studies of the gastrointestinal effects (i.e., effects on motility) are warranted and necessary.

## Data Availability

The raw data supporting the conclusions of this article will be made available by the authors, without undue reservation upon request.

## Footnotes

^1^ www.randomizer.org
