# Supplementary material for: Pharmacokinetics and thermal anti-nociceptive effects of oral morphine in horses
Source: Front Vet Sci. 2024 Sep 17;11:1461648. doi: 10.3389/fvets.2024.1461648 (PMC11443510; doi:10.3389/fvets.2024.1461648)
Supplement: Supplementary file 1 [file Supplementary_file_1.docx]

**Supplementary Information:**

***Determination of plasma morphine, morphine-6-glucuronide, and morphine-3-glucuronide***

The analytical reference standards for morphine, morphine-6-glucuronide (M6G), and morphine-3-glucuronide (M3G) were obtained from Cerilliant (Round Rock, TX) as 1 mg/mL solutions, and the internal standard D3-M3G was obtained as a 0.1 mg/mL solution. Acetonitrile (ACN) (Burdick and Jackson, Muskegon, MI), water (Burdick and Jackson, Muskegon, MI), methanol (Fisher Scientific, Fair Lawn, NJ), buffer reagents (Fisher Scientific, Fair Lawn, NJ) and formic Acid, 97% (Fisher Scientific, Fair Lawn, NJ) were HPLC grade or better.

Morphine, M3G and M6G were all combined into one working solution which were prepared by dilution of the 1 mg/mL stock solutions with methanol to concentrations of 10, 100, 1000, and 10,000 ng/mL. Plasma calibrators were prepared by dilution of the working standard solutions with drug free plasma to concentrations of 0.25, 0.5, 1, 5, 10, 25, 50, 100, 150, 200, 300, 400, 500, 600, 700, and 800 ng/mL. Calibrators with concentrations of 900, 1000, and 1100 ng/mL were added for M3G quantitation. Calibration curves and negative control samples were prepared fresh for each quantitative assay. In addition, quality control samples (drug free equine plasma fortified with analyte at four concentrations (0.75, 35, 160 and 600 ng/mL) within the standard curve) were included with each sample set as a check of accuracy.

Prior to analysis, 500 µL of plasma was diluted with 500 µL of ACN:1M Acetic Acid (9:1, v:v) containing 50 ng/mL of D3- M3G internal standard, to precipitate proteins. The samples were vortexed for 2 minutes to mix, refrigerated for 20 minutes, vortexed for an additional one minute, centrifuged (4300 rpm/3102 g) for 10 minutes at 4ºC and 500 µL of the organic layer transferred to a tube and dried under nitrogen at 55 ºC. Samples were dissolved in 150 µL of 5% ACN in water, with 0.2% formic acid, and 30 µL was injected into the Liquid Chromatography-Mass Spectrometry (LC-MS/MS) system.

The concentration of morphine, M6G, and M3G were measured in plasma by LC-MS/MS using positive heated electrospray ionization (HESI(+)). Quantitative analysis of plasma was performed on a TSQ Vantage triple quadrupole mass spectrometer (Thermo Scientific, San Jose, CA) coupled with a turbulent flow chromatography system (TFC TLX4) (Thermo Scientific, San Jose, CA) having 1100 series liquid chromatography systems (Thermo Scientific, San Jose, CA) and operated in laminar flow mode. The spray voltage was 3000V, the vaporizer temperature was 300ºC, and the sheath and auxiliary gas were 40 and 20 respectively (arbitrary units). Product masses and collision energies of each analyte were optimized by infusing the standards into the TSQ Vantage. Chromatography employed a Zorbax Eclipse-XDB-Phenyl 2.1x150mm, 5μm column (Agilent Technologies, Palo Alto, CA) and a linear gradient of ACN in water with a constant 0.2% formic acid at a flow rate of 0.4 ml/min. The initial ACN concentration was held at 0% for 0.67 minutes, ramped to 60% over 5 minutes and ramped to 95% over 0.5 minutes before re-equilibrating for 3.08 minutes at initial conditions.

Detection and quantification were conducted using Selective Reaction Monitoring (SRM) of initial precursor ion for morphine (mass to charge ratio 286.189 (*m/z)*), M3G (mass to charge ratio 462.185 (*m/z)*), M6G (mass to charge ratio 462.185 (*m/z)*), and the internal standard D3-M3G (mass to charge ratio 465.188 (*m/z)*). Retention times were used to distinguish between the M3G and M6G metabolites. The response for the product ions for morphine (*m/z* 153.1, 165.1, 201.2), M3G (*m/z* 165, 286.2), M6G (*m/z* 165, 286.2) and the internal standard D3-M3G (*m/z* 165, 289.2) were plotted and peaks at the proper retention time integrated using Quanbrowser software (Thermo Scientific, San Jose, CA). The software was used to generate calibration curves and quantitate morphine, M3G, and M6G in all samples by linear regression analysis. A weighting factor of 1/X was used for all calibration curves.

**Supplementary Tables:**

**Supplementary Table 1**. Accuracy and Precision Values for LC-MS/MS analysis of morphine, morphine 6-glucuronide (M6G) and morphine 3-glucuronide (M3G) in equine plasma.

| Analyte | Concentration  (ng/mL) | Accuracy (% nominal concentration) | Precision  (% relative SD) |
| --- | --- | --- | --- |
| Morphine |  |  |  |
|  | 0.75 | 106 | 4.0 |
|  | 40.0 | 106 | 2.0 |
|  | 250 | 98.0 | 2.0 |
| M6G |  |  |  |
|  | 0.75 | 92.0 | 4.0 |
|  | 40.0 | 100 | 2.0 |
|  | 250 | 103 | 2.0 |
|  |  |  |  |
|  |  |  |  |
| M3G | 0.75 | 94.0 | 5.0 |
|  | 40.0 | 103 | 3.0 |
|  | 250 | 100 | 2.0 |
