# Supplementary material for: Pharmacokinetics and thermal anti-nociceptive effects of oral morphine in horses
Source: Front Vet Sci. 2024 Sep 17;11:1461648. doi: 10.3389/fvets.2024.1461648 (PMC11443510; doi:10.3389/fvets.2024.1461648)
Supplement: Supplementary file 2 [file Image_1.pdf]

A.) **B.)**

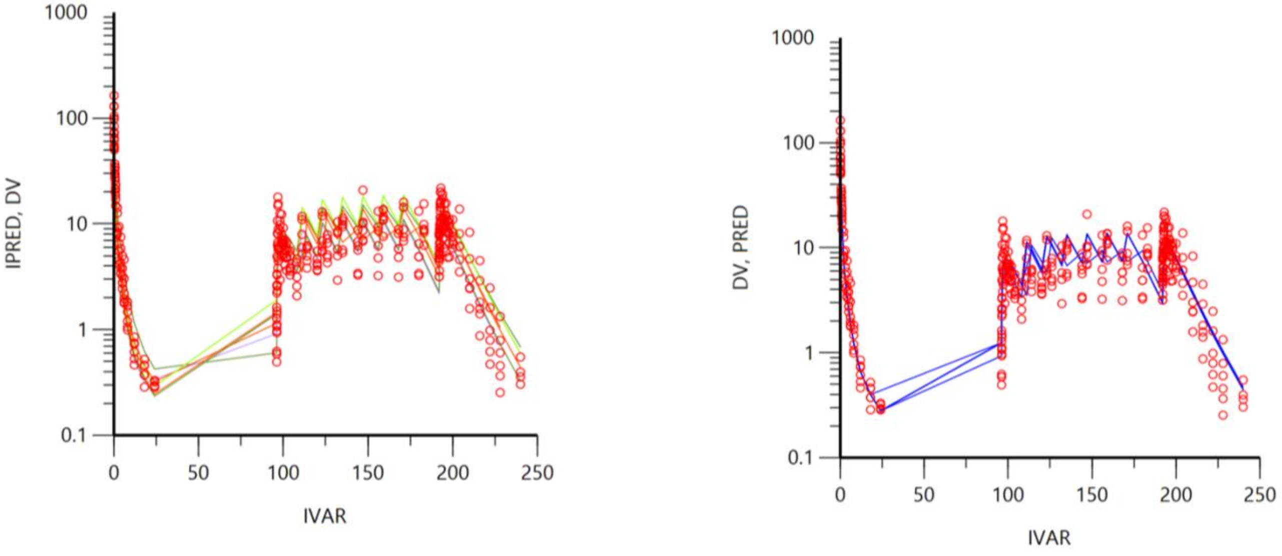

Supplementary Figure 1. (A) Plot of dependent variable (DV: concentrations) and individual prediction (IPRED) vs independent variable (IVAR; time) (B) Plot of dependent variable (DV; concentrations) and population prediction (PRED) vs the independent variable (IVAR; time).

A.) **B.)**

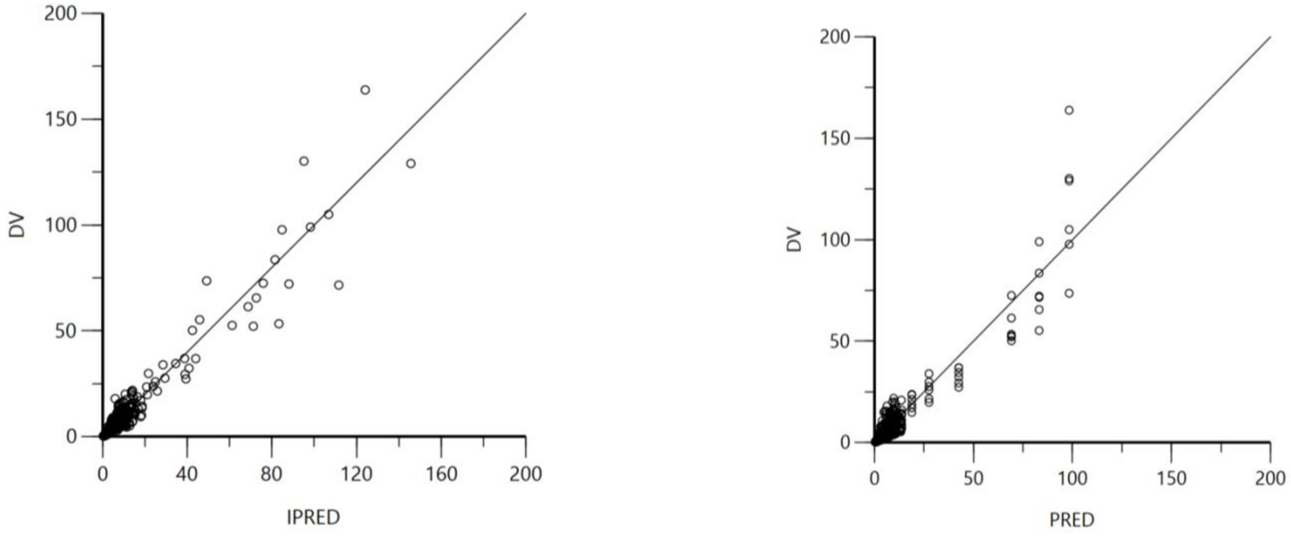

Supplementary Figure 2. (A) Plot of the dependent variable (DV: concentrations) versus individual predicted values (IPRED; predicted concentrations) and (B) Plot of the dependent variable (DV; concentrations) versus population predications (PRED).
